# Pulmonary Embolism as a Finding During Endobronchial Ultrasound: An Occasional Occurrence or a New Element to Be Staged?

**DOI:** 10.7759/cureus.20137

**Published:** 2021-12-03

**Authors:** Alberto Fantin, Nadia Castaldo, Benjamin Seides, Maria Majori

**Affiliations:** 1 Department of Pulmonology, University Hospital of Udine (ASUFC), Udine, ITA; 2 Department of Infectious diseases, University Hospital of Udine (ASUFC), Udine, ITA; 3 Canning Thoracic Institute, Northwestern Medicine, Chicago, USA; 4 Internal Medicine - Pulmonology, University Hospital of Parma, Parma, ITA

**Keywords:** staging, pulmonary artery, nsclc, pulmonary emboli, ebus

## Abstract

In this report, we describe two cases of lung cancer-related pulmonary embolism (PE), both encountered while performing an endobronchial ultrasound (EBUS). We propose EBUS as a diagnostic and confirmatory method for PE detection during the staging of lung cancer.

## Introduction

Pulmonary embolism (PE) associated with lung cancer is a known entity, and lung cancer itself is a known risk factor. Still, its diagnosis during bronchoscopy with endobronchial ultrasound (EBUS) is highly unusual [1]. PE can be a life-threatening condition clinically manifesting as acute dyspnoea, chest pain, palpitations, or syncope. The literature already contains evidence that supports the use of EBUS in order to confirm an endovascular lesion and determine the differential diagnosis between the invasion of a lung or vessel tumor or pure thromboembolism [2].

## Case presentation

Case 1

**Figure 1:**
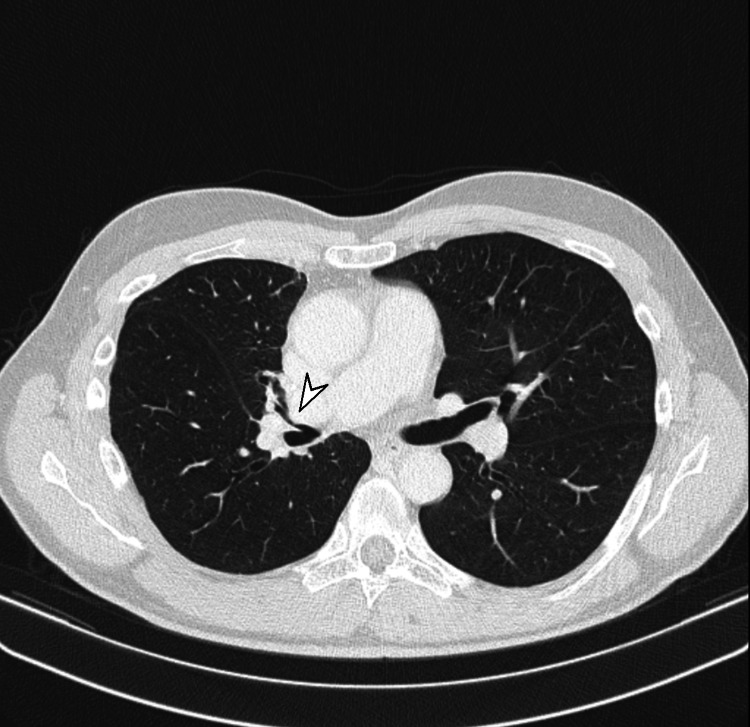
Chest CT showing an upper right lobar bronchus stenosis (white arrowhead) CT: computed tomography

**Figure 2:**
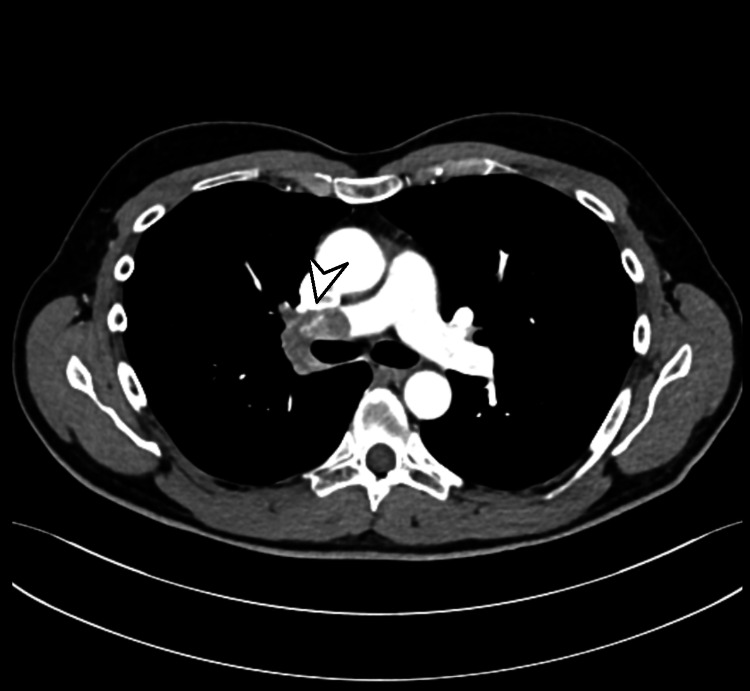
Chest CT showing infiltration of the main neoplastic lesion into the right pulmonary artery (white arrowhead) CT: computed tomography

A 61-year-old male, asthmatic, and a non-smoker, was initially evaluated as an outpatient for chronic cough. He had experienced chronic exposure to dust due to his bricklaying work. A chest X-ray showed hilar enlargement. The CT of the thorax (Figure 1) demonstrated a mixed stenotic-compressive lesion in right the upper lobe bronchus, secondary to a hilar mass that was in close contact with the wall of the ipsilateral pulmonary arterial vessels. The CT also demonstrated the presence of a unilateral proximal PE contiguous to the primary pulmonary lesion (Figure 2). The presence of significant lymphadenopathy in the 4R station was simultaneously demonstrated.

**Figure 3:**
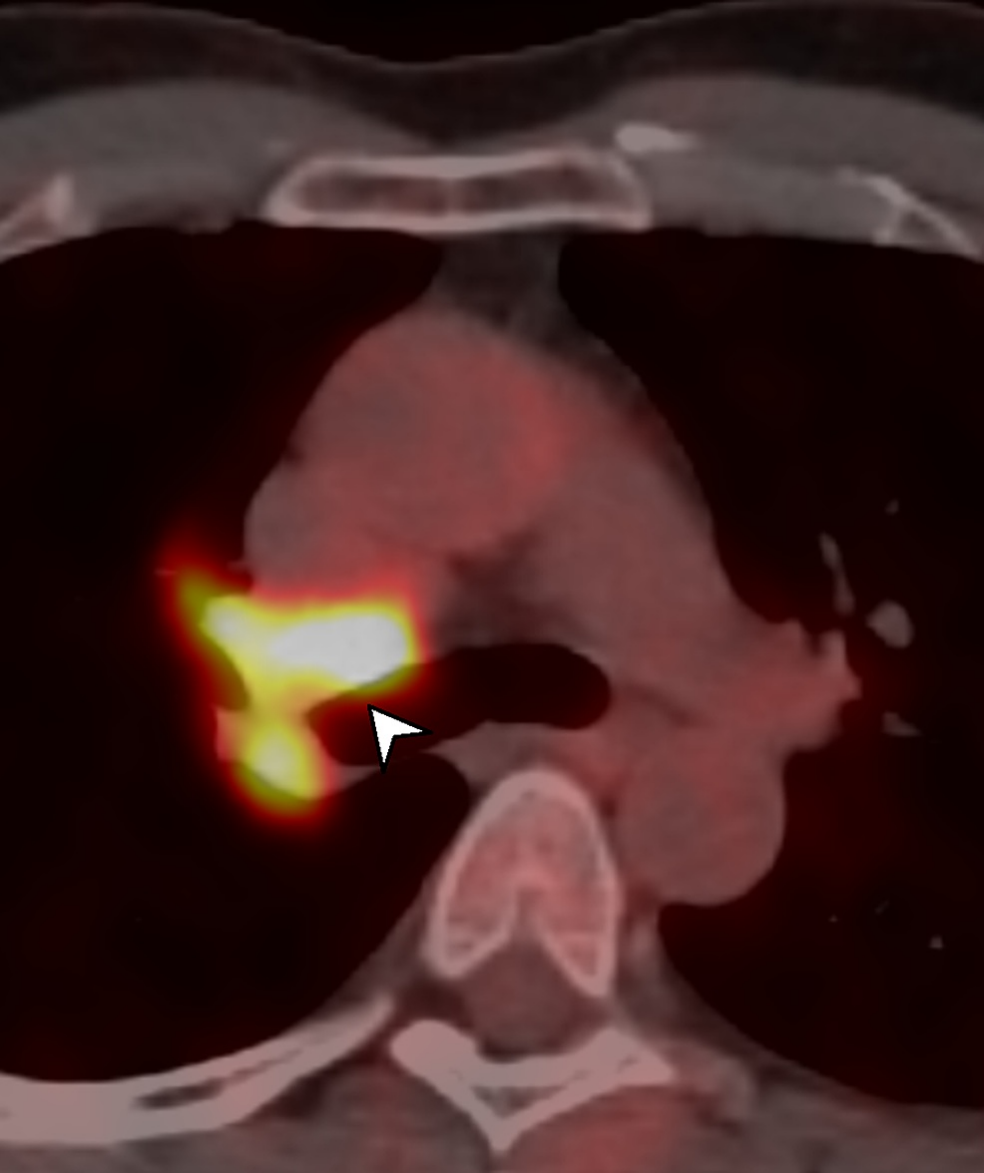
PET-CT showing a hypermetabolic lesion (white arrowhead) PET-CT: positron emission tomography-computed tomography

A PET-CT (Figure 3) was performed, which demonstrated an area of intense hyperaccumulation of 18FDG [standardized uptake value (SUV): 19.4] due to the known solid lesion to the right lung hilum. An intense accumulation of contrast medium was also found at the level of mediastinal lymphadenopathy in the 4R station.

No deep vein thrombosis was found on the lower extremity Doppler ultrasound. Cardiac ultrasound showed a non-dilated left ventricle with preserved wall thicknesses. The global systolic function of the left ventricle was preserved in the absence of gross alterations in segmental kinetics. The Vd-Ad gradient was 15 mmHg; the inferior vena cava was not dilated and collapsed in deep inspiration. Pulmonary artery pressure (PAP) was estimated at 20 mmHg. The patient was given anticoagulant therapy and managed on an outpatient basis.

**Figure 4:**
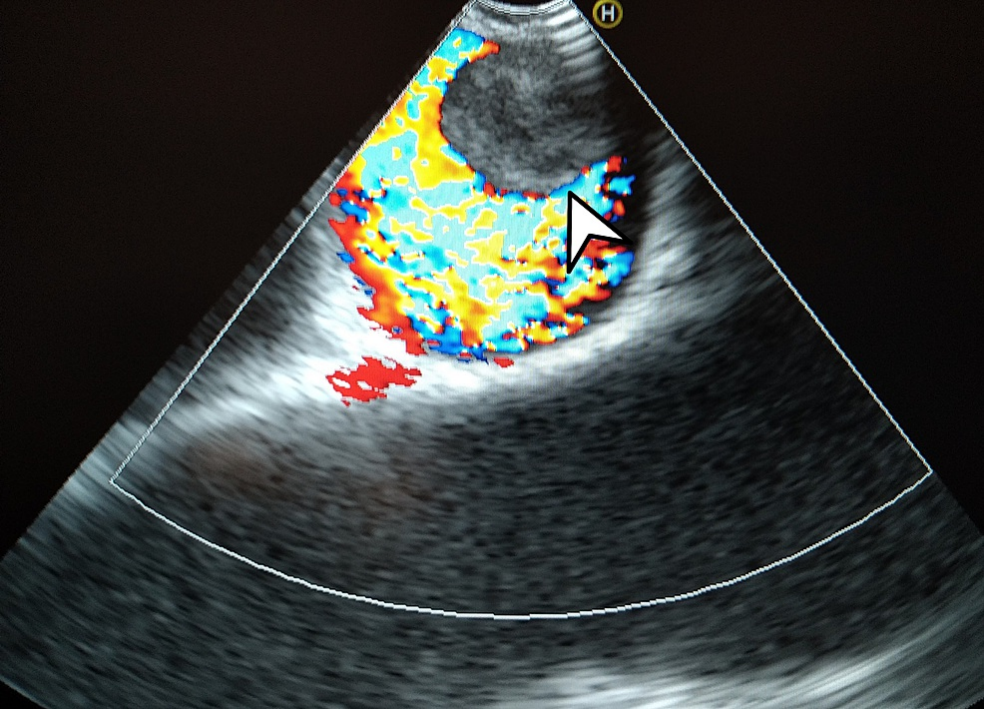
Color Doppler showing the blood flow around the intravascular tumor

A bronchoscopy with selective EBUS-guided transbronchial needle aspiration (TBNA) was then performed. The procedure showed an exophytic lesion in the right upper bronchus, which was sampled by endobronchial biopsy. The EBUS examination revealed lymph nodes of less than 5 mm in diameter in the 4L, 7, 10L, and 11L stations and a crescentic 16-mm intravascular opacity in the 10R station, raising suspicion for a tumor or clot thrombus in the pulmonary artery (PA) (Figure 4). Four passes were taken at the 4R station. The sampling of the endobronchial mass revealed an adenocarcinoma of the lung.

The tumor demonstrated a favorable anaplastic lymphoma kinase (ALK) translocation. After a multidisciplinary team discussion, given the favorable lung function tests, and after neoadjuvant therapy with alectinib, the patient was surgically treated with radical intent by right thoracotomy, with right upper lobectomy.

Case 2

**Figure 5:**
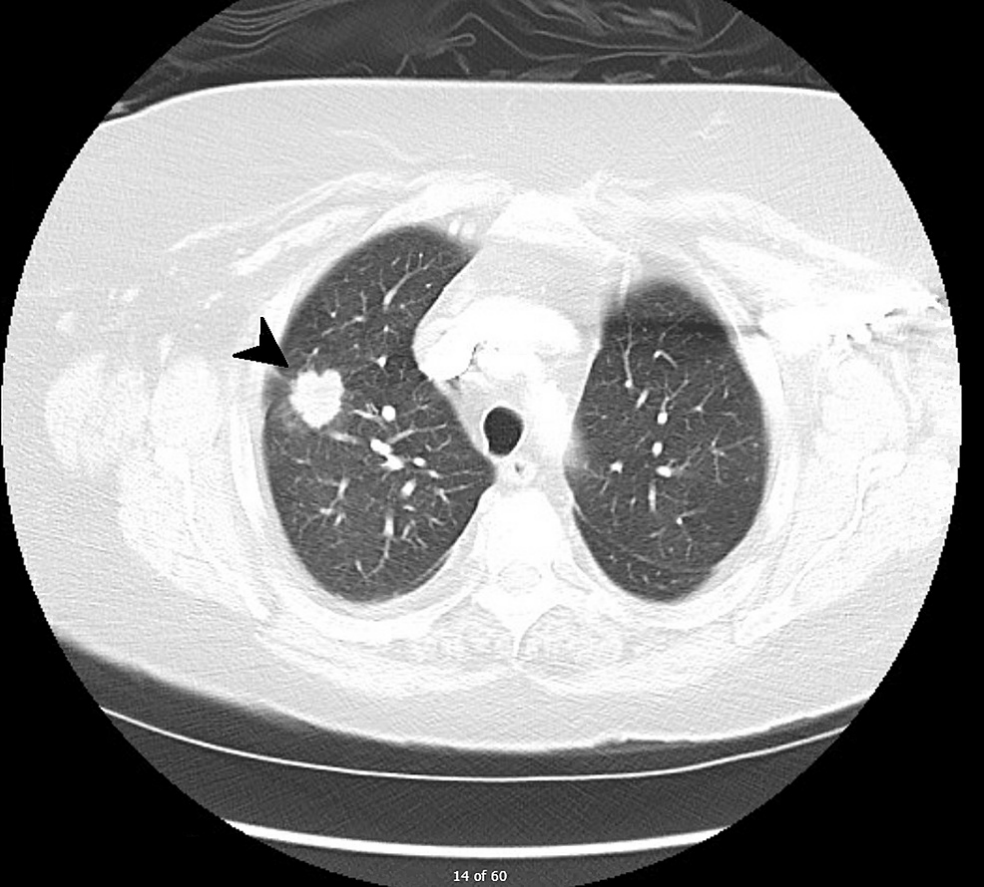
Chest CT showing an upper right lobe nodule (black arrowhead) CT: computed tomography

A 72-year-old female, a 40 pack-year smoker, was screened for lung cancer with low-dose chest CT (Figure 5), which revealed the presence of a spiculated lung nodule in the right upper lobe. The presence of significant lymphadenopathy in the 4R station was simultaneously demonstrated.

**Figure 6:**
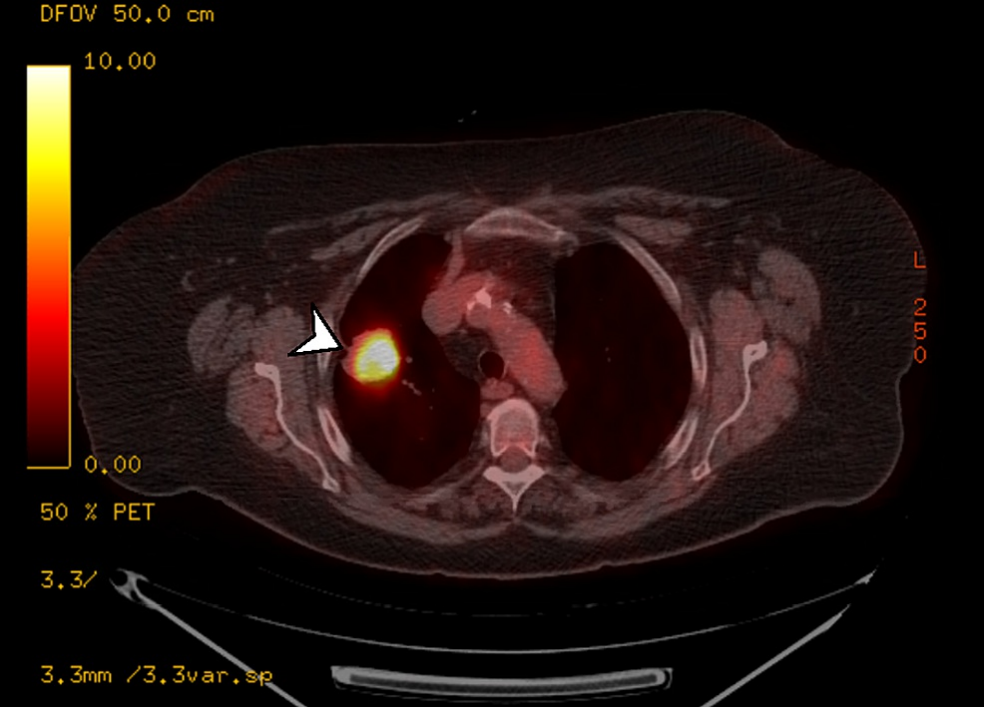
PET-CT showing the hypermetabolic state of the nodule (white arrowhead) PET-CT: positron emission tomography-computed tomography

She was initially referred by her primary doctor for percutaneous lung biopsy by the interventional radiology team, which revealed squamous cell lung cancer. She was then lost to follow-up for several months due to the coronavirus disease 2019 (COVID-19) crisis and eventually presented again for care. Re-staging of the disease was considered a major priority, and a PET-CT was performed (Figure 6). The known squamous cell tumor was hypermetabolic on imaging (SUV: 13.3), while the 4R lymph nodes previously identified on tomography were not.

**Figure 7:**
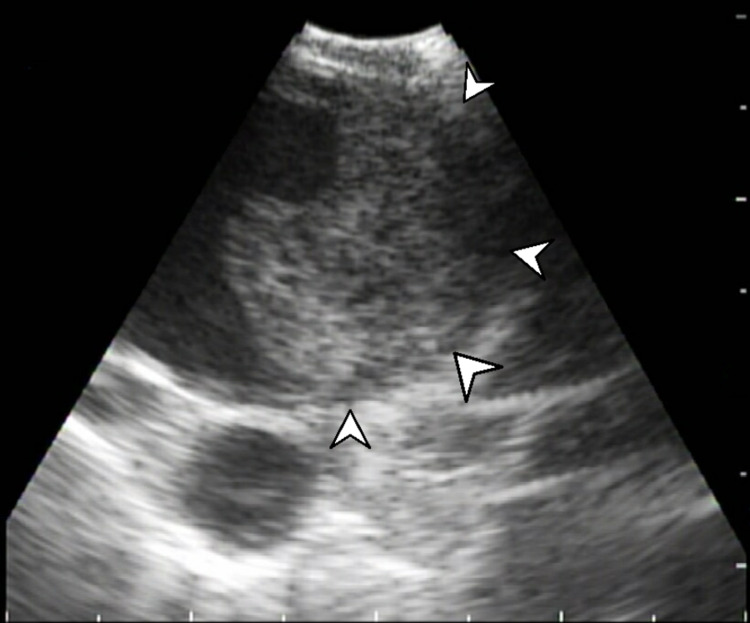
EBUS imaging of the 11R station, demonstrating a newly discovered pulmonary embolism (white arrowheads) EBUS: endobronchial ultrasound

**Figure 8:**
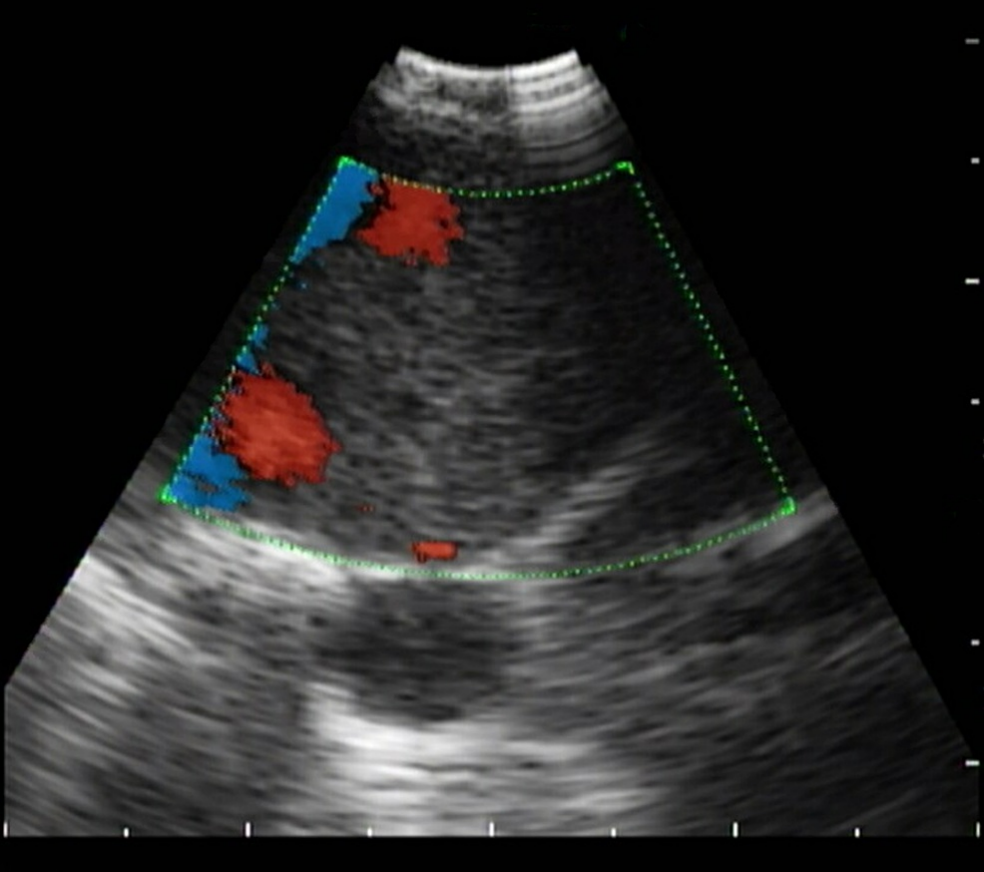
Color Doppler showing the blood flow around the embolus

She underwent bronchoscopy with mediastinal staging with EBUS. A systematic ultrasound examination revealed an 8-mm lymph node in the 11L left interlobar station, insignificant lymph node(s) in the subcarinal location, insignificant lymph node(s) in the 4L left paratracheal location, 15-mm lymph node(s) in the 4R right paratracheal location, and a crescentic 15-mm opacity, intravascularly located in the 11R right interlobar location (Figures 7, 8). Four passes were taken at the station 11L location, and three passes were taken at the station 4R location. Upon careful review of the imaging, no biopsies were taken of the 11R lesion, as it appeared to have an intravascular location, raising the question of tumor or clot thrombus in the PA.

**Figure 9:**
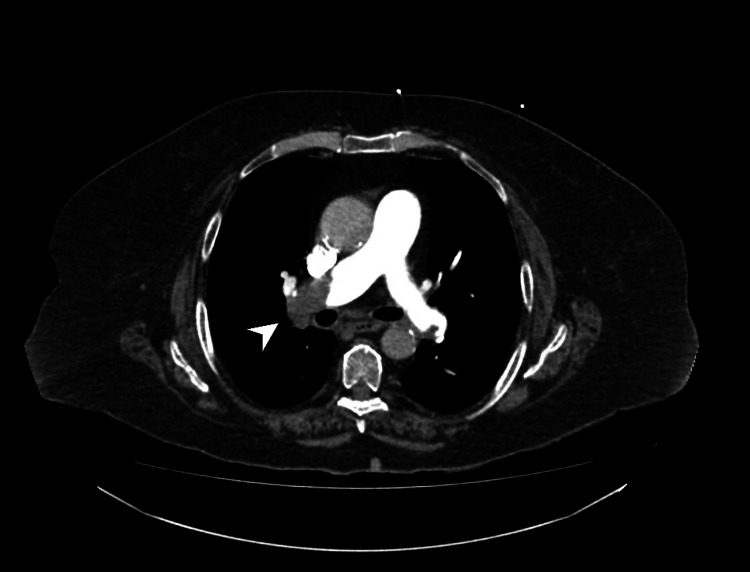
Chest CT confirming acute pulmonary embolism (white arrowhead) CT: computed tomography

At the end of the procedure, the patient was sent to the emergency room for a CT angiogram of the chest, which revealed bilateral PE (Figure 9). No deep vein thrombosis was found on the lower extremity Doppler ultrasound. A cardiac ultrasound revealed evidence of right heart strain. She was started on a novel oral anticoagulant and discharged home.

Subsequently, it was determined that she was not a surgical candidate based on pulmonary function tests and was therefore referred for curative stereotactic body radiotherapy.

## Discussion

We described two different cases in terms of presentation and time to diagnosis of PE related to the bronchoscopic procedure and therapeutic approach.

The first patient began his diagnostic process because of an asymptomatic issue and was referred after undergoing a thoracic CT with a contrast medium. The second case was referred following the patient's participation in a lung cancer screening program, which by protocol is performed without a contrast medium.

We observed some differences between the cases even in terms of the nature of the pulmonary embolic lesion. In the first case, the intravascular material was PET-avid, denoting a hypermetabolic state, typically associated with an intravascular neoplastic lesion [3]. In the second case, the clinical picture was more explicable as a PE associated with a hypercoagulable state in a patient with lung cancer. While CT cannot definitively differentiate between the tumor or non-tumor nature of the embolic lesions, EBUS could provide this differentiation by evaluating the contiguity between the main lesion and the intravascular mass, observing the different echogenicity, and strain and vascular patterns. TBNA sampling of the endovascular lesion is also technically feasible; however, the risks associated with this procedure must be carefully considered [4].

The timing of the finding also differed between the two patients. In the first case, the intravascular lesion was found on pre-procedural imaging, while in the second one, the procedure prompted the evaluation of the patient in the ER for excluding an acute PE. Hence, we propose that a contrast-enhanced thoracic CT could be requested before every procedure aimed at staging a suspected malignant lesion, in order to evaluate the mediastinum and study the vascular structures with respect to nearby organs.

The discussion regarding the finding of PE, alongside the evaluation of the other peculiarities found on thoracic imaging, should be conducted by a multidisciplinary group involving thoracic oncology. In this setting, given the anesthesiological risk factors, a tailored therapeutic approach must be planned in order to maximize the chances of patient survival and reduce both morbidity and mortality [5].

## Conclusions

Based on the observations reported in this case series, we believe that vascular evaluation while performing EBUS should be considered as a new step in a systematic approach, as both vascular invasion and concomitant PE could alter the patient's therapeutic management path. Due to the anticipated establishment of lung cancer screening as a standard of care in the future, many patients with suspicious lesions would be referred to interventional pulmonology units for both diagnosis and staging requirements. These patients will not undergo a contrast-enhanced CT to guide the interventions. We need to be aware of missing this important diagnosis that would significantly alter the prognosis of our patients.

In conclusion, we suggest that every bronchoscopist evaluate, with ultrasound and color Doppler, all the accessible vessels during EBUS, to check for intravascular lesions that are either known to exist before the procedure or to screen for new ones.
